# Biologically Targeted Magnetic Hyperthermia: Potential and Limitations

**DOI:** 10.3389/fphar.2018.00831

**Published:** 2018-08-02

**Authors:** David Chang, May Lim, Jeroen A. C. M. Goos, Ruirui Qiao, Yun Yee Ng, Friederike M. Mansfeld, Michael Jackson, Thomas P. Davis, Maria Kavallaris

**Affiliations:** ^1^Children's Cancer Institute, Lowy Cancer Research Centre, University of New South Wales, Sydney, NSW, Australia; ^2^Department of Radiation Oncology, Nelune Comprehensive Cancer Centre, Prince of Wales Hospital, Sydney, NSW, Australia; ^3^ARC Centre of Excellence in Convergent Bio-Nano Science and Technology and Australian Centre for Nanomedicine, University of New South Wales, Sydney, NSW, Australia; ^4^School of Chemical Engineering, University of New South Wales, Sydney, NSW, Australia; ^5^ARC Centre of Excellence in Convergent Bio-Nano Science and Technology, Monash Institute of Pharmaceutical Sciences, Monash University, Melbourne, VIC, Australia; ^6^Department of Radiology, Memorial Sloan Kettering Cancer Center, New York, NY, United States; ^7^Department of Chemistry, University of Warwick, Coventry, United Kingdom

**Keywords:** magnetic hyperthermia, targeted therapy, iron oxide nanoparticles, cancer therapy, magnetic nanoparticles

## Abstract

Hyperthermia, the mild elevation of temperature to 40–43°C, can induce cancer cell death and enhance the effects of radiotherapy and chemotherapy. However, achievement of its full potential as a clinically relevant treatment modality has been restricted by its inability to effectively and preferentially heat malignant cells. The limited spatial resolution may be circumvented by the intravenous administration of cancer-targeting magnetic nanoparticles that accumulate in the tumor, followed by the application of an alternating magnetic field to raise the temperature of the nanoparticles located in the tumor tissue. This targeted approach enables preferential heating of malignant cancer cells whilst sparing the surrounding normal tissue, potentially improving the effectiveness and safety of hyperthermia. Despite promising results in preclinical studies, there are numerous challenges that must be addressed before this technique can progress to the clinic. This review discusses these challenges and highlights the current understanding of targeted magnetic hyperthermia.

## Introduction

Hyperthermia, a treatment aimed at raising the temperature of cancerous regions of the body to 40–43°C, can induce cancer cell death by enhancing the cytotoxic effects of radiotherapy and chemotherapy (Wust et al., 2002). Extensive preclinical and clinical research into the application of hyperthermia has been conducted, with a number of randomized trials demonstrating that, when combined with radiotherapy, it has the potential to improve the outcomes of various cancers without significantly increasing toxicity (De Haas-Kock et al., 2009; Lutgens et al., 2010). Despite these promising results, hyperthermia is rarely incorporated into modern oncological management due to its ineffectiveness when applied as a single modality treatment and a lack of large phase 3 clinical trials combining hyperthermia with both standard chemotherapy and radiotherapy (van der Zee, 2002). Furthermore, a major disadvantage of conventional hyperthermia is that, in general, both malignant and non-malignant cells are equally sensitive to heating (Dewey et al., 1977; Roizin-Towle and Pirro, 1991). This is in contrast to chemotherapy or radiotherapy, which are generally more cytotoxic toward malignant cells. As a result, there has been significant interest in the concept of “biologically targeted magnetic hyperthermia,” whereby targeted magnetic iron oxide nanoparticles (MIONs) are administered intravenously in order to heat tumors under an alternating magnetic field. In this review, we will discuss the current understanding of targeted magnetic hyperthermia and the limitations that must be overcome for further progression into clinical practice.

## Hyperthermia and cell death

**Figure 1:**
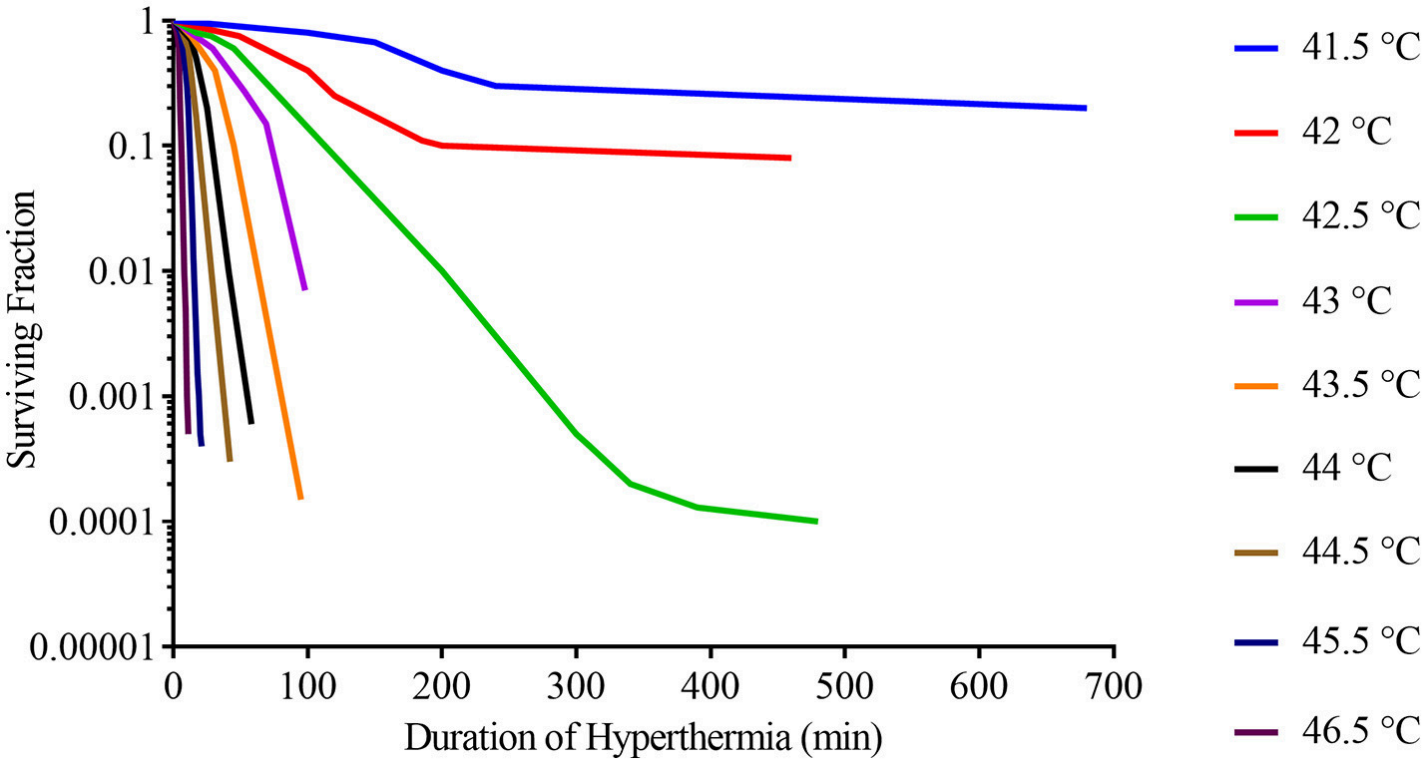
Survival curves for asynchronous Chinese hamster ovary (CHO) cells heated at different temperatures for varying lengths of time. Adapted from Dewey et al. (1977).

Hyperthermia can cause cell death through a range of different mechanisms and there are no consistent differences in thermal sensitivities between malignant and non-malignant cells (Dewey et al., 1977; Roizin-Towle and Pirro, 1991). It has been shown *in vitro* that cell viability following hyperthermia treatment is heavily influenced by both the temperature and the duration of hyperthermia (Figure 1). Even half a degree rise in temperature can have a substantial impact on cell viability, highlighting the importance of effective and homogenous delivery of hyperthermia (Dewey et al., 1977). One of the possible mechanisms behind the reduction in cell viability is protein denaturation with subsequent activation and deactivation of several downstream pathways (van der Zee, 2002; Wust et al., 2002). Individual proteins have specific temperature thresholds for denaturation, with highly expressed proteins generally being more tolerant to heat (Leuenberger et al., 2017). Protein denaturation occurs from approximately 40°C and higher temperatures will denature a greater proportion of proteins, which may explain why the rate of cell death rises with the temperature (Lepock, 2005b). At temperatures of 40–42°C, only a small fraction of proteins is denatured, however, some of these can subsequently co-aggregate with native proteins, thereby significantly increasing the level of aggregation (Borrelli et al., 1996). It is this combination of heat-induced denaturation and subsequent co-aggregation that is thought to affect several downstream pathways including inactivation of protein synthesis, cell cycle progression and DNA repair (Dewey et al., 1977; Kampinga et al., 2004; Lepock, 2005a). Furthermore, possibly through a mechanism that is unrelated to protein denaturation, hyperthermia can have an adverse impact on the cytoskeleton, organelles, intracellular transport, and RNA processing (Richter et al., 2010). Another potential contributor to reduction in cell viability is heat-induced alterations in the plasma and subcellular organelle membranes, as well as membrane proteins (Richter et al., 2010; Mello et al., 2017).

Sufficient application of hyperthermia can result in cell death (Figure 1), but if cells survive several major classes of proteins will be activated leading to thermotolerance. These classes of proteins include: heat shock proteins that stabilize misfolded proteins, proteolytic enzymes that clear denatured/aggregated proteins, RNA-, and DNA-modifying proteins that repair damage, and others (Richter et al., 2010).

In addition to the responses to hyperthermia at a cellular level described above, hyperthermia may impart its effects via several additional, unique mechanisms on cell communities and these have been investigated *in vivo*. Tumors are generally associated with hypoxic and acidic environments due to poor vasculature, conditions in which cells are known to be more susceptible to hyperthermia (Gerweck et al., 1979; Eales et al., 2016). Elevated temperatures can lead to increased perfusion within the tumor, leading to greater chemotherapeutic drug delivery and higher oxygen concentrations, which in turn can sensitize tumors to radiotherapy (Song et al., 1996; Rau et al., 2000). Hyperthermia may enhance the immune response via several mechanisms, including increased migration of immune effector cells to the tumor, modulation of cell surface molecules and various pro-inflammatory cytokines, proliferation of effector cells, and increased immune cell cytotoxicity against malignant cells (Peer et al., 2010).

Despite the multitude of mechanisms by which hyperthermia can induce cell death, it is not efficient as a single agent treatment, mainly due to its poor specificity and the development of thermotolerance which may make subsequent hyperthermia treatments less effective. However, in combination with radiotherapy or chemotherapy, hyperthermia can lead to improved patient outcomes.

## Hyperthermia in combination with radiotherapy and chemotherapy

In the clinic, hyperthermia can be applied to a local area, a specific region of the body or the entire body. In the past few decades, mild elevations of temperature have been achieved by various means including thermal chambers, hot water blankets, application of electromagnetic energy, perfusion of limb or body cavity with heated fluids, ultrasound and MIONs (van der Zee, 2002; Wust et al., 2002). In order to improve the efficacy, hyperthermia has often been evaluated as an adjunct treatment to enhance radiotherapy and cytotoxic chemotherapy. One way of expressing the enhancement of radiotherapy or chemotherapy is via the thermal enhancement ratio (TER), where TER is the ratio of the dose of radiation or drug alone that is required to achieve the end point to the dose of radiation or drug combined with heat to achieve the same end point (Overgaard, 1984). As an example, 60 min of hyperthermia at 42°C, can result in a TER of nearly 2 for radiotherapy, making hyperthermia one of the most potent radiosensitizers (Overgaard, 1984). Hyperthermia is thought to enhance radiotherapy via protein denaturation and the subsequent inactivation of proteins involved in DNA repair. Inactivation of DNA repair proteins, particularly those involved in excision of clustered base damage, may prevent repair of the DNA damage induced by radiotherapy, leading to increased cell death (Kampinga and Dikomey, 2001). *In vivo*, hyperthermia can prime the tumor to radiotherapy via increased vascular perfusion and oxygenation of previously radioresistant, hypoxic areas (Song et al., 2005). Both preclinical and clinical evidence indicates that the TER is highest when hyperthermia is delivered simultaneously or in close temporal proximity to radiotherapy when protein denaturation and aggregation are likely to be at their greatest (van Leeuwen et al., 2017). Furthermore, the TER increases with temperature and duration of hyperthermia (Overgaard, 1984).

Hyperthermia can synergistically enhance the efficacy of numerous chemotherapeutic agents including cisplatin, cyclophosphamide and bleomycin, whilst no significant enhancement for 5-fluorouracil, doxorubicin, and vincristine has been observed. For example, the application of 30 min of hyperthermia at 41.5°C *in vivo*, can result in a TER of 1.48 for cisplatin and 2.28 for cyclophosphamide (Urano et al., 1999). Although the exact mechanism for chemosensitization is poorly understood, for alkylating or alkylating-like platinum agents like cyclophosphamide and cisplatin, their ability to interact with and encourage protein denaturation may be partly responsible (Lepock, 2005b). *In vivo*, hyperthermia can lead to chemosensitivity via increased tumor blood flow and increased vascular permeability resulting in increased accumulation of chemotherapeutic agent (Song et al., 2005).

There have been a number of randomized clinical trials on the impact of hyperthermia on various cancers in combination with radiotherapy or chemotherapy or both (Tables 1–3), with many other studies currently in progress (Valdagni et al., 1988; Berdov and Menteshashvili, 1990; Datta et al., 1990; Sharma et al., 1991; Sugimachi et al., 1994; Kitamura et al., 1995; Overgaard et al., 1996; Vernon et al., 1996; Sneed et al., 1998; Harima et al., 2001; van der Zee, 2002; Jones et al., 2005; Franckena et al., 2008; Verwaal et al., 2008; Huilgol et al., 2010; Issels et al., 2010; Colombo et al., 2011; Cihoric et al., 2015; Arends et al., 2016). The majority of studies demonstrated higher rates of local response with only mild to moderate toxicities. It is worth noting that there is some heterogeneity in the outcomes, which may be due to differences in heating protocols. An area of deficiency, and perhaps one of the reasons why hyperthermia is rarely used in the clinic is that delivering sufficient hyperthermia to the tumor, whilst sparing the surrounding normal tissue, is difficult.

**Table 1:** List of randomized clinical trials on hyperthermia combined with radiotherapy.

**Reference**	**Cancer type**	**Number of patients randomized**	**Type of treatment**	**Outcomes**	**Toxicity from hyperthermia**
Valdagni et al., 1988	Fixed and inoperable N3 cervical nodal squamous cell carcinoma metastases from either a previous, concomitant T1-T3 head and neck primary or unknown primary	44 nodes	Control arm: Radiotherapy Experimental arm: Radiotherapy + hyperthermia (radiative hyperthermia, 280-300 MHz within 20–25 min of irradiation, ≥42.5°C for 30 min, 2–6 treatments)	Complete response rates: 82.3% for experimental arm and 36.8% for control arm *p* = 0.0152 Thermal enhancement ratio = 2.23	Similar acute toxicities between control and experimental arm
Datta et al., 1990	Head and neck carcinoma Stage I-IV	65	Control arm: Radiotherapy Experimental arm: Radiotherapy + hyperthermia (capacitive hyperthermia, 27.12 MHz, immediately before radiotherapy, ≥42.5°C for 20 min, twice a week)	At 18 months post treatment, 19% disease free survival for control and 33% for experimental arm *p* = 0.11 For stages III and IV, control 8%, experimental 25% *p =* 0.03, 79% of study group had almost complete alleviation of pain compared to only 50% of control group p < 0.02	3 of 33 patients in the experimental arm developed local erythema and facial edema
Berdov and Menteshashvili, 1990	T4N0M0 Rectal carcinoma	115	Control arm: Pre-operative radiotherapy Experimental arm: Pre-operative radiotherapy and hyperthermia (capacitive hyperthermia involving an endorectal antenna, 915 MHz, 42-43°C for 1 h, 4–5 treatments, radiation delivered within 10 min)	55.4% of experimental arm were able to have an operation compared to 27.1% for control arm 5 year survival 35.6% for experimental arm compared to 6.6% for control group p < 0.05	Comparable post-operative complications between control and experimental arm
Sharma et al., 1991	Stage II and III Cervical Carcinoma	50	Control arm: Radiotherapy Experimental arm: Radiotherapy + hyperthermia (capacitive hyperthermia involving an intravaginal electrode, 27.12 MHz, 42-43°C for 30 min, radiation delivered within 30 min, 3 times per week for 4 weeks)	18 months of follow-up Local control 50% for control arm 70% for experimental arm p < 0.05	No major toxicity from hyperthermia
Perez et al., 1991	Superficial Tumors	245	Control arm: Radiotherapy Experimental arm: Radiotherapy + hyperthermia (radiative hyperthermia, 915 MHz, 43°C for 60 min immediately after irradiation, 8 treatments)	Improved local control for tumors <3cm but not for tumors >3cm	30% incidence of thermal blisters in the experimental arm
Vernon et al., 1996^*^	Patients with advanced primary or recurrent breast cancer having local radiotherapy rather than surgery	306	Control arm: Radiotherapy Experimental arm: Radiotherapy + hyperthermia (via various devices and frequencies depending on the study location, ≥42.5°C for ≥30 min, various intervals between radiotherapy and hyperthermia, 2-8 treatments)	Complete response for the control arm 41% 59% for hyperthermia arm p < 0.001 Greatest difference seen in patients with recurrent lesions in previously irradiated areas, where further irradiation was limited to low dose	More acute toxicities in the experimental arm: Blisters: 11% vs. 2% Ulceration 7% vs. 2% Necrosis 7% vs. 1% Comparable rates of late toxicity between the control and experimental arm
Overgaard et al., 1996	Recurrent or metastatic malignant melanoma	134 lesions in 70 patients	Control arm: Radiotherapy Experimental arm: Radiotherapy + hyperthermia (variable mode of delivery, hyperthermia delivered within 30 min of radiotherapy, aimed for >60 equivalent minutes of 43°C but in reality only a median of 9 equivalent minutes of 43°C achieved, 3 treatments)	Complete response rate 62% for experimental arm and 35% for radiotherapy only control arm *p =* 0.003	Similar acute or late radiation reactions in control and experimental arm
Emami et al., 1996	Persistent or recurrent tumors after previous radiotherapy and/or surgery, amenable to interstitial radiotherapy	171	Control arm: Interstitial radiotherapy Experimental arm: Interstitial radiotherapy + hyperthermia (delivered by either 300-2450 MHz microwave antennas or 0.1-1 MHz radiofrequency currents, ≥43°C for 60 min, hyperthermia delivered within 60 min of irradiation, 1-2 sessions)	No difference in survival or complete response.	Similar toxicity between control and experimental arm
Van Der Zee et al., 2000	Muscle-invasive bladder cancer (including T2, T3, T4, N0, M0) Cervical Cancer Stages IIB, IIIB or IV Rectal Cancer Stage M0-M1	361	Control arm: Radiotherapy Experimental arm: Radiotherapy + hyperthermia (delivered using various systems, 42°C for 60 min, within 1–4 h after radiotherapy, 5 treatments)	Complete response rates: 39% control arm 55% experimental arm p < 0.001 Lower local failure rate for hyperthermia arm: (relative hazard ratio 0.76) *p =* 0.04 At 3 years, no significant difference in overall survival except for cervical cancer (51% and 27% *p =* 0.009)	Cases of burns in the experimental arm Similar rates of late radiation toxic effects between control and experimental arm
Harima et al., 2001	Stage IIIB cervical carcinoma	40	Control arm: External beam radiotherapy + high dose rate intracavitary brachytherapy Experimental arm: External beam radiotherapy + high dose rate intracavitary brachytherapy + hyperthermia (capacitive heating device, 8 MHz, delivered within 30 min of radiotherapy, for a total of 60 min, average temperature of 40.6°C achieved, 3 sessions)	Significant difference in 3-year local relapse-free survival 48.5% control arm 79.7% experimental arm *p =* 0.048 No significant improvement in 3-year overall survival and disease-free survival	Similar rates of acute or late toxicity between the control and experimental arm
Jones et al., 2005	Malignancy ≤3 cm in thickness from the body surface	109	All patients received hyperthermia (radiative hyperthermia, 433 MHz, for ≤ 1 h maximum allowable temperature of normal tissue 43°C) for 1 h. If they were unable to achieve a thermal dose of ≥0.5 CEM 43°C T90, they were not randomized. Rest of patients were then randomized. Control: No further hyperthermia but had radiotherapy Experimental: Hyperthermia + radiotherapy (twice a week, 1–2 h, targeted between 10-100 cumulative equivalent minutes at 43°C T90)	Complete response rate: Hyperthermia arm 66% Control arm 42% *p =* 0.02 Note that some patients received systemic treatment but there was no significant difference in the proportion of patients in each arm who received systemic therapy No significant difference in overall survival	Grade 1 and 2 thermal burns 41% in experimental arm 4% in control arm Grade 3 thermal burns 5% for experimental arm 2% in control arm 11% catheter (used to monitor the temperature) related side effects for experimental arm 2% for control arm
Franckena et al., 2008	Locoregionally advanced cervical cancer	114	Control arm: Radiotherapy Experimental arm: Radiotherapy + hyperthermia (via various systems depending on site, >42°C for 60 min, 5 treatments)	12 year follow-up Local control: 37% for hyperthermia arm 56% for control *p =* 0.01	Similar rates of late toxicity between control and experimental arm
Huilgol et al., 2010	T2-T4, N0-N3, M0 Oropharynx, hypopharynx or oral cavity carcinoma	56	Control Arm: Radiotherapy Experimental Arm: Radiotherapy + hyperthermia (via capacitive system, 8.2MHz, power increased until patients complained of discomfort, power reduced and treatment continued for 30 min, 5-7 sessions)	Statistically significant difference in median survival of control group 145 days Experimental group 241 days	Comparable acute and late toxicities between control and experimental arm, except for overall increase in thermal burns in the experimental arm

* *Meta-analysis of 5 randomized trials. The 5 trials were not published separately due to slow accrual*.

**Table 2:** List of randomized clinical trials on hyperthermia combined with chemotherapy.

**Reference**	**Cancer type**	**Number of patients randomized**	**Type of treatment**	**Outcomes**	**Toxicity from hyperthermia**
Ghussen et al., 1984	Malignant melanoma of the extremities	107	Control arm: Local excision and regional lymph node dissection Experimental arm: Local excision and regional lymph node dissection + hyperthermia perfusion (via extracorporal heating of heparinized whole blood, limb temperatures were elevated to 42°C, 60 min) with melphalan (added once limb temperature reached ≥40°C)	Significant improvement in disease-free survival *p =* 0.0001 Significant improvement in survival. *p =* 0.0207	Higher rates of reversible post-operative complications in the experimental arm
Hafström et al., 1991	Recurrent malignant melanoma of the extremities	69	Control arm: Surgery Experimental arm: Surgery + regional hyperthermic perfusion (via extracorporeal heating of blood mixed with low molecular weight dextran and heparin, temperature of the inflow perfusate was maintained at 41.5–41.8°C, maintained for 1 h, melphalan added either beginning or at the end of hyperthermic perfusion)	Improved tumor-free survival *p =* 0.044 Difference in median survival not statistically significant	Higher rates of post-operative complications in the experimental arm
Hamazoe et al., 1994	Gastric cancer with gross serosal invasion but no gross peritoneal metastasis	82	Control arm: Surgery Experimental arm: Surgery + continuous hyperthermic peritoneal perfusion with mitomycin C (after gastrectomy, saline containing mitomycin C was heated and infused into the peritoneal cavity via silicon tubes, inflow termperature was maintained between 44–45°C, 50–60 min)	No statistically significant difference in overall survival.	Higher rates of transient abnormal blood profiles after surgery in the experimental arm
Sugimachi et al., 1994	Thoracic esophageal squamous cell carcinoma	40	Control arm: Chemotherapy +/- Oesophagectomy Experimental arm: + hyperthermia (via capacitive system involving an endotract electrode, 42.5–44°C for 30 min, 6 sessions) ^+/−^ Oesophagectomy	Subjective improvement of dysphagia: 40% in control arm vs. 70% for experimental arm Radiographic improvement: 25% in control arm and 50% in experimental arm Histological response: 18.8% in control arm vs. 58.3% in experimental arm *p* < 0 0.05	Similar rates of toxicity between control and experimental arm
Koops et al., 1998	Primary cutaneous melanoma at high risk of having regional micrometastases	832	Control: Wide excision Experimental arm: Wide excision and isolated limb perfusion with melphalan and mild hyperthermia (limb was perfused heated perfusate, maintaining tissue temperatures of 39–40°C for 60 min, melphalan delivered once subcutaneous temperature reached 38°C)	No survival benefit	Higher rates of transient post-operative toxicity in the experimental arm
Verwaal et al., 2008	Peritoneal carcinomatosis of colorectal cancer	105	Control: Chemotherapy (5-fluorouracil, leucovorin weekly for 26 weeks or until progression or unacceptable toxicity. If treated with 5-fluorouracil within 12 months before randomization, received irinotecan at 3 weekly intervals for 6 months, or until progression or intolerable toxicity) + surgery (only if symptoms of intestinal obstruction). Experimental arm: Cytoreductive surgery, intra-operative hyperthermic intraperitoneal chemotherapy (initial warming via >3 l isotonic dialysis fluid, at 1–2 l/min and an inflow temperature of 41–42°C for 90 min, Mitomycin C added once abdominal temperature stable at 40°C) + adjuvant systemic chemotherapy.	Median follow-up of almost 8 years Median progression-free survival: 7.7 months for control arm and 12.6 months in hyperthermia arm *p =* 0.02 Median disease-specific survival: 12.6 months in control arm and 22.2 months in hyperthermia arm *p =* 0.028	Toxicity higher for experimental arm including 3 of 54 patients in the experimental arm dying from abdominal sepsis
Colombo et al., 2011	Intermediate to high-risk non-muscle invasive bladder cancer	83	Control arm: Transurethral resection and 2 doses of mitomycin C Experimental arm: Transurethral resection and 2 doses of mitomycin C + hyperthermia (via a 915 MHz intravesical radiative hyperthermia device, median temperature of 42 ± 2°C for ≥40 min, 8 x weekly and 4 x monthly sessions)	Median follow-up 91 months 10-year disease-free survival: 53% with thermochemotherapy 15% with chemotherapy *p* < 0.001	Similar rates of acute and late toxicity between control and experimental arm
Arends et al., 2016	Intermediate to high risk non-muscle-invasive bladder cancer	190	Control Arm: Bacillus Calmette-Guerin immunotherapy Experimental arm: 6 × weekly mitomycin C + 6 × 6-weekly maintenance mitomycin C and hyperthermia (via a 915 MHz intravesical radiative hyperthermia device, 42 ± 2°C, 60 min, 6 x weekly sessions followed 6 further treatments at 6 week intervals)	24 month recurrence free survival was 81.8% in experimental arm and 64.8% in the control arm *p =* 0.02	Mitomycin C + Hyperthermia group associated with less urinary frequency, nocturia, incontinence, hematuria, fever, fatigue and arthralgia but more catheterisation difficulties, urethral strictures, bladder tissue reaction, bladder spasms, bladder pain, allergies

**Table 3:** List of randomized clinical trials on hyperthermia combined with radiotherapy and chemotherapy.

**Reference**	**Cancer type**	**Number of patients randomized**	**Type of treatment**	**Outcomes**	**Toxicity from hyperthermia**
Kitamura et al., 1995	Squamous cell carcinoma of the thoracic esophagus undergoing neoadjuvant therapy	66	Control arm: Neoadjuvant chemoradiotherapy + surgery Experimental arm: Neoadjuvant hyperthermochemoradiotherapy (capacitive system involving an intraluminal applicator, 42.5–44°C at tumor surface for 30 min, 6 sessions)	Complete response 25% in experimental arm 5.9% in control arm 3 year survival 50.4% experimental arm 24.2% control arm	Details lacking No postoperative mortality in either arm
Sneed et al., 1998	Glioblastoma	79	Control arm: Radiotherapy + oral hydroxyurea + brachytherapy boost Experimental arm: Radiotherapy + oral hydroxyurea + brachytherapy boost + hyperthermia (radiative hyperthermia, 915 MHz, ≥42.5°C for 30 min, 15–30 min before and after brachytherapy)	Median survival: 76 weeks for control arm 85 weeks for hyperthermia arm *p =* 0.02	There was a trend (*p =* 0.08) toward more grade 3 or higher toxicities for the experimental arm Higher incidence of grade 1 and grade 2 neurological changes and seizures for the experimental arm
Issels et al., 2010	Localised high-risk soft-tissue sarcoma, extremity and retroperitoneal	341	Control arm: Neoadjuvant and adjuvant chemotherapy (etoposide, ifosfamide, doxorubicine) + local therapy (surgery +/- radiotherapy) Experimental arm: Neoadjuvant and adjuvant chemotherapy (etoposide, ifosfamide, doxorubicine) + local therapy (surgery +/- radiotherapy) + regional hyperthermia (radiative hyperthermia, 42°C for 60 min on day 1 and 4 of 3 weekly chemotherapy cycles, up to 8 sessions)	Median follow-up 34 months Significant improvement in local progression-free survival (hazard ratio = 0.58, *p* = 0.003) and disease-free survival (hazard ratio = 0.7, *p* = 0.011)	Increased pain, bolus pressure, skin burn in experimental arm

## Magnetic hyperthermia

Despite the ability of hyperthermia to enhance radio- and chemotherapy treatments, toxicity due to the similar responses of malignant, and healthy tissues to hyperthermia remains a barrier to clinical application. A promising approach to overcoming this obstacle is magnetic hyperthermia, a form of hyperthermia that is currently undergoing clinical trials. It was first proposed by Gilchrist et al. (1957), who introduced the concept of injecting MIONs (20–100 nm), into lymphatic channels in order to heat residual cancer cells under an Alternating Magnetic Field (AMF) (Gilchrist et al., 1957). In 1993, Jordan et al. showed that delivering magnetic nanoparticles via direct injection into the tumor could result in much more effective and selective heating of tumors when compared to other heating techniques such as radiofrequency heating and ultrasound (Jordan et al., 1993). Furthermore, there is *in vitro* evidence that certain types of cancers including glioblastoma cells can take up magnetic nanoparticles more efficiently than non-malignant cells, although the exact mechanism is not well understood (Jordan et al., 1999). Since then, significant efforts have gone into the development of a clinical AMF system, resulting in the formation of a publicly listed company, MagForce AG based in Germany. The company has developed NanoTherm® aminosilane coated ferrofluid, NanoActivator® alternating magnetic field applicator, and NanoPlan® temperature simulation software.

In the past 2 decades, phase 1, and 2 clinical studies of intratumorally delivered magnetic nanoparticles and the subsequent application of AMF via the MagForce system have been successfully conducted for patients with glioblastoma and prostate cancers (Johannsen et al., 2005, 2007a,b; Maier-Hauff et al., 2011). Phase 1 clinical studies on patients with prostate cancer demonstrated the feasibility of the approach with no significant late treatment-related morbidity. The average temperatures achieved were in the hyperthermic range (40–43°C), as opposed to the thermoablative range (>50°C). Although there were PSA declines following magnetic hyperthermia, responses in the monotherapy trial were of limited extent and duration and therefore, a phase 2 trial is now recruiting patients with intermediate risk prostate cancer and is evaluating magnetic hyperthermia in combination with low dose rate brachytherapy (Johannsen et al., 2010). Furthermore, MagForce has recently received an Investigational Device Exemption (IDE) for use in patients with intermediate prostate cancer undergoing active surveillance. Recruitment of patients with intermediate risk prostate cancer will commence after approval by ethics committees (Magforce, 2013, 2018). It is hoped that hyperthermia treatment in such patients can control the more aggressive component of the tumor and prevent or delay the need for radiotherapy or surgery. A phase 2 clinical trial involving 66 patients with recurrent glioblastoma, demonstrated a median overall survival of 13.4 months from the time of tumor recurrence (Maier-Hauff et al., 2011). Acute toxicities observed in this study included tachycardia (18.2%), headaches (13.6%), motor disturbances (21.2%), and convulsions (22.7%), which may be prevented with anti-epileptic drugs. In the magnetic hyperthermia study, however, no prolonged side effects were observed other than worsening motor disturbances, which may be related to disease progression rather than magnetic hyperthermia (Maier-Hauff et al., 2011). Following the phase 2 clinical trial, MagForce has been conducting a randomized, controlled trial (DRKS00005476) to determine the efficacy and safety of NanoTherm® monotherapy and NanoTherm® in combination with radiotherapy vs. radiotherapy alone in recurrent/progressive glioblastoma. The study is now closed and the final report of the data will be submitted to the official bodies this year (Magforce, 2013, 2018).

## Magnetic iron oxide nanoparticles for magnetic hyperthermia

The most commonly used materials for magnetic hyperthermia are nanometre size (10–100nm) ferrite nanoparticles, in particular magnetite (Fe_3_O_4_) or maghemite (γ-Fe_2_O_3_). Fe_3_O_4_ and γ-Fe_2_O_3_ are commonly and collectively referred to as MIONs. The magnetic properties of MIONs arise from the presence of ions with different valency in their crystal structure. For instance, Fe_3_O_4_ consists of two trivalent iron (III) ions and one divalent iron (II) ion. The unpaired ions result in parallel but oppositely aligned magnetic moments that do not cancel out and thus are subject to strong, spontaneous magnetization.

**Figure 2:**
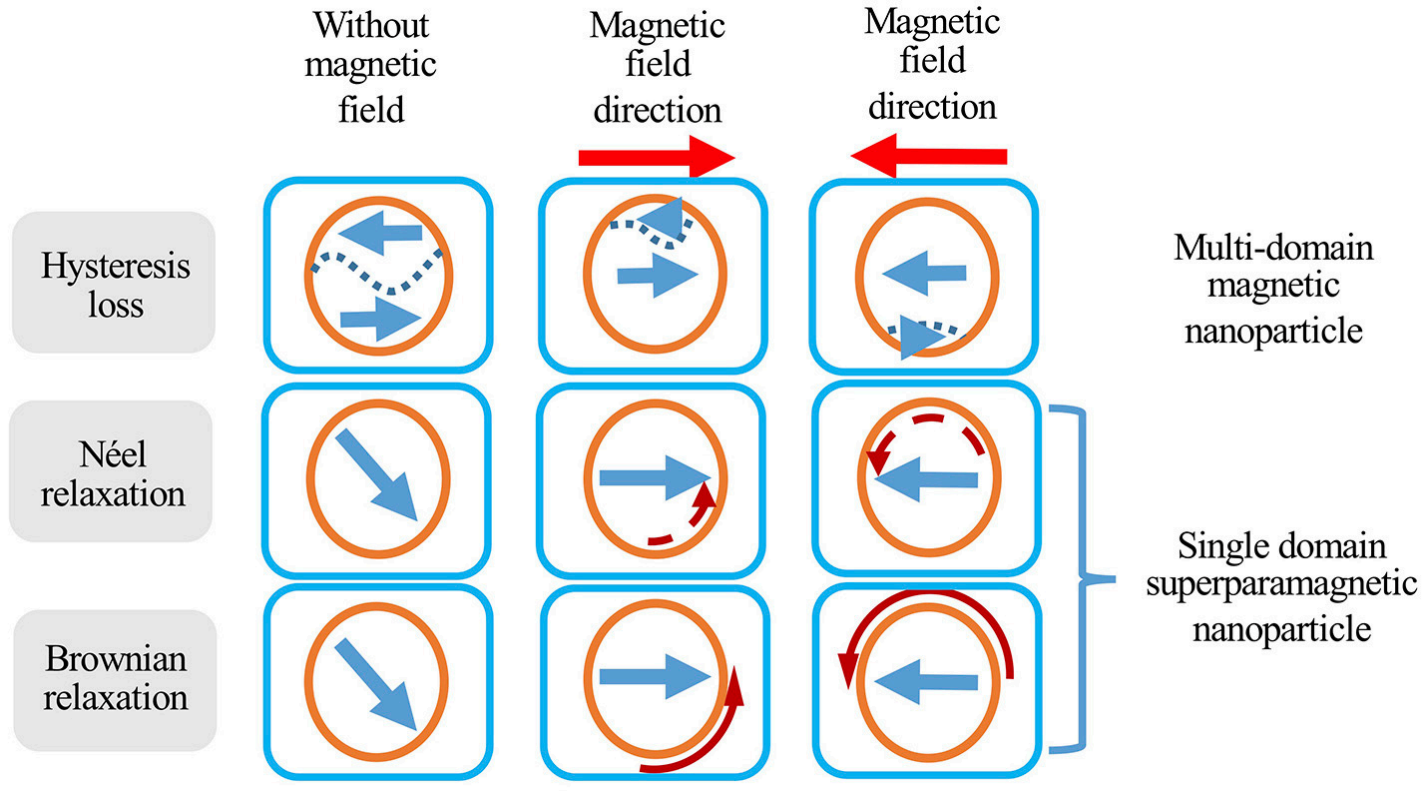
Different heat generation mechanisms of magnetic nanoparticles in response to an alternating magnetic field. Orange circles represent MIONs, short straight arrows represent magnetic field direction, curved arrows represent the movement (solid curved arrow) or change in magnetic moment direction (dashed curved arrow), and dashed lines represent domain boundaries in multi-domain particles. Adapted from Suriyanto et al. (2017).

When exposed to an alternating magnetic field, MIONs produce heat via two main mechanisms: (1) hysteresis loss and (2) relaxational losses. Hysteresis losses occur in large MIONs which possess multiple magnetic domains. When such particles are subjected to an alternating magnetic field, the orientation of the magnetic moments will align continuously with the direction of the magnetic field as illustrated in Figure 2. This results in a difference in energy that is released in the form of heat (Kirschning et al., 2012). As MION size decreases, the number of magnetic domains will also decrease until a single magnetic domain remains at a threshold size of approximately 128 nm (Houlding and Rebrov, 2012). Below this size, MIONs are deemed superparamagnetic and in the presence of an AMF, heat is mainly produced by Néel relaxation and Brownian relaxation. Néel relaxation refers to rapid changes in the particle's magnetic moment when exposed to AMF (Figure 2). The rapid realignment is opposed by the particle's crystalline structure, resulting in heat generation. Brownian relaxation refers to the frictional heat generated from the physical rotation of particles within a supporting medium when the particles attempt to realign themselves with the changing magnetic field (Figure 2; Suto et al., 2009; Suriyanto et al., 2017). A more comprehensive discussion on the mechanism of heating is beyond the scope of this review and covered elsewhere (Ruta et al., 2015).

MIONs have the advantage of long term chemical stability and biocompatibility, and ease of surface modification and functionalisation when compared to other types of magnetic susceptible materials such as certain metals (e.g., iron, nickel or cobalt) or metal alloys (e.g., FePt, FeCo), (Dunn et al., 2014). Furthermore, MIONs can act as a contrast agent for computed tomography (CT) at high concentrations and magnetic resonance imaging (MRI) at lower concentrations, with several iron oxide nanoparticles previously approved by the FDA for these applications (Anselmo and Mitragotri, 2015). This is particularly useful since the concentration of the MIONs within the tumor can be estimated via CT and this can aid the estimation of hyperthermia dosimetry (Johannsen et al., 2007b). MIONs have also been shown to enhance the effects of radiotherapy even in the absence of AMF, potentially by increasing the generation of reactive oxygen species (ROS) through the Fenton reaction (Huang et al., 2010; Klein et al., 2012; Khoei et al., 2014; Bouras et al., 2015). Finally, iron is an essential component of the human body and the average human adult naturally carries approximately 3.5–4 grams of iron. Consequently, unlike other inorganic nanoparticles, MIONs have been systemically delivered safely in large quantities in clinical settings (Hetzel et al., 2014). Furthermore, there is *in vitro* evidence that intracellular localized heating of ligand-decorated MIONS can lead to lysosomal damage of the target cells and induce cell death even in the absence of bulk heating (Creixell et al., 2011; Domenech et al., 2013).

## Mode of delivery

**Figure 3:**
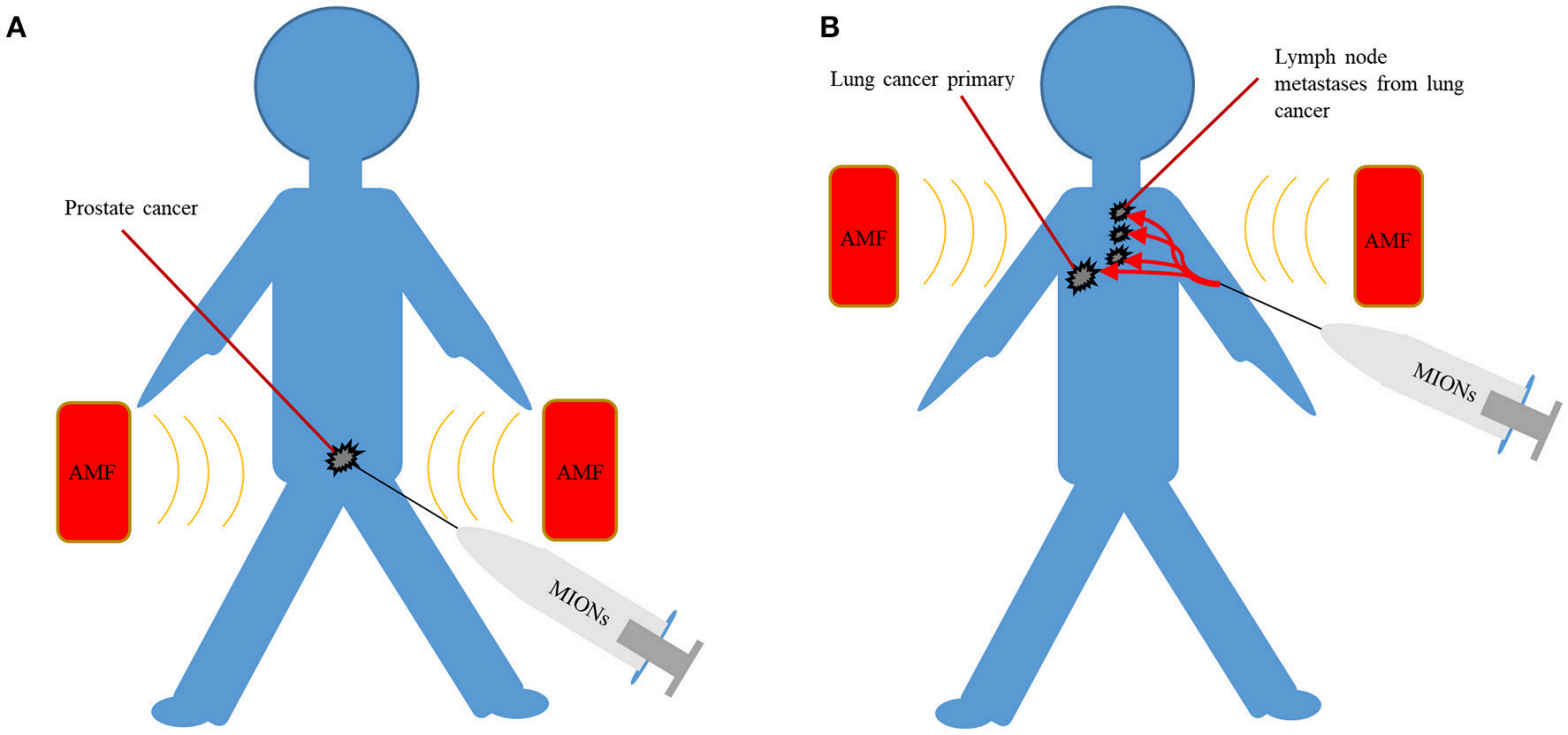
**(A)** Intra-tumoral delivery can achieve high concentrations of MIONs but are only suited to localized disease such as prostate cancer. **(B)** Intravenous delivery can potentially target poorly localized malignancies, often with lymph node metastases, such as lung cancer. AMF, alternating magnetic field; MIONs, magnetic iron-oxide nanoparticles.

MIONs can potentially be delivered to the tumor via intra-tumoral, intra-peritoneal, intra-arterial, intra-cavitary, and intravenous administration. Oral administration of MIONs is not feasible as most of the nanoparticles will be fecally excreted, owning to their large size (Chamorro et al., 2015). Intra-tumoral administration of MIONs efficiently localizes MIONs in the tumor and can result in effective heating of primary tumors such as prostate cancer. Intra-tumoral administration can result in very high concentrations of MIONs within the tumor and can remain localized in the tumor. When MIONs were directly injected to the prostate in men with localized prostate cancer, MIONs were still clearly visible on CT 6 weeks post injection, thereby allowing repeated magnetic hyperthermia treatments (Johannsen et al., 2005). In a separate post-mortem study of patients with glioblastoma who received MIONs, nanoparticles were restricted to the site of intra-tumoral injection, once again confirming a good retention profile (Van Landeghem et al., 2009). However, intra-tumoral delivery of MIONs is not practical for larger tumors with regional metastases and is more invasive than other techniques (Figure 3). Furthermore, poorly defined tumors like GBM may be better targeted by intravenously delivered MIONs which are less dependent on the operator for effective delivery, although penetrating the blood-brain barrier may be a challenge.

Intra-peritoneal mode of delivery is well suited to cancers that often spread to the peritoneal cavity such as ovarian, pancreatic and gastric cancers. Cancer targeting MIONs have been successfully delivered via the intra-peritoneal route and have demonstrated significant uptake by both primary and metastatic tumors in orthotopic mouse pancreatic cancer models (Gao et al., 2017). When compared to intravenous mode of delivery, the intra-peritoneal route achieved an intra-tumoral level that was 3-fold higher. The same system was able to carry chemotherapeutic drugs and significantly inhibited pancreatic tumors without systemic toxicity (Gao et al., 2017). Toraya-Brown et al. administered non-targeted MIONs intra-peritoneally in an aggressive mouse metastatic ovarian cancer model and demonstrated significant accumulation of MIONs in the tumor (Toraya-Brown et al., 2013). They determined that the non-targeted MIONs were taken up by peritoneal phagocytes and delivered to tumors. When under an AMF, MIONs generated enough heat to induce cell death within tumors. A separate mouse study determined that up to 5 mg/kg of MIONs can be safely delivered intraperitoneally although at higher levels, signs of oxidative damage were detected within the hepatic and renal tissues (Ma et al., 2012). Furthermore, monocyte/macrophage-like cells with a propensity to migrate into tumors, can be loaded with MIONs externally and injected intraperitoneally, after which the cells will direct MIONs for magnetic hyperthermia (Basel et al., 2012).

For bladder cancers, magnetic hyperthermia can be achieved by the direct injection of MIONs into the bladder cavity via a urinary catheter. The thick lining of the bladder will restrict the absorption of MIONs and once the treatment is completed, MIONs can be removed through the catheter, thereby minimizing systemic toxicity. The feasibility of this approach was demonstrated by Oliveira et al. in rat bladders, where temperatures of 42°C were maintained in the bladder with minimal heating of surrounding tissues (Oliveira et al., 2013).

Intra-arterial administration of chemotherapeutic drugs has been successfully applied to liver cancers in the clinic. MIONs may be well suited to this task as they tend to accumulate in the liver via the reticuloendothelial system. With this in mind, the arterial delivery of iron oxide nanoparticles has been explored in preclinical models by several investigators (Lee et al., 2013, 2017; Kim et al., 2016). In rabbits, when MIONs were delivered with iodized oil and doxorubicin, there was an increased intra-tumoral accumulation of drugs and consequently, reduced numbers of viable tumor cells (Lee et al., 2013). For lung cancer, there have been early investigations into the potential formulation of aerosolized MIONs and their delivery via a combination of a nebulizer and a magnet (Dames et al., 2007; Tewes et al., 2014; Graczyk et al., 2015). However, it would be quite challenging to deliver sufficient quantities of MIONs for the application of magnetic hyperthermia using this approach.

Although the modes of administration mentioned so far are well suited to particular scenarios, intravenous administration is the most versatile method of delivery for the widest range of cancers. Not surprisingly, intravenous delivery is one of the most common routes of administration of chemotherapeutic drugs and in the past, FDA-approved MION MRI contrast agents have been delivered intravenously. (Figure 3). When MIONs are delivered in this manner, the accumulation of nanoparticles within the tumor depends in part on the enhanced permeability and retention (EPR) effect (Iyer et al., 2006). The EPR effect refers to the tendency of nanoparticles to preferentially accumulate within tumors due to their leaky vasculatures and poor lymphatic drainage. Once nanoparticles have reached the tumor, targeting ligands, such as small molecules, peptides or antibodies, bound to MIONs may lead to increased association and uptake of nanoparticles by malignant cells (DeNardo et al., 2007; Balivada et al., 2010). Their preferential accumulation within the malignant cells can lead to targeted heating of tumors and sparing of adjacent normal tissue under AMF (DeNardo et al., 2007; Balivada et al., 2010). Such approaches may potentially result in more homogeneous delivery of MIONs to the tumor and would be far less operator dependent when compared to other forms of targeting. In addition, the avoidance of the surgical morbidity associated with intra-tumoral injection of MIONs in the tumor may be attractive.

So far, preclinial *in vivo* studies (Table 4) have been performed in order to demonstrate the concept of biologically targeted magnetic hyperthermia (Table 4). Huang et al. intravenously injected extremely large quantities (1,700 mg Fe/kg) of untargeted MIONs into mice and achieved a subcutaneous tumor concentration of 1.9 mg Fe/kg of tumor. Despite the lack of targeting moiety, they were able to achieve a tumor to surrounding non-tumor concentration ratio of more than 16:1 via the enhanced permeability and retention effect alone. Application of AMF achieved significant tumor control when compared to either nanoparticles or AMF alone. In the same study, mice injected with even higher doses of nanoparticles (3,400 mg Fe/kg) survived more than 12 months without showing any clinical signs of toxicity (Huang and Hainfeld, 2013). Another *in vivo* study on intravenous administration of porphyrin coated MIONs demonstrated improved melanoma tumor control under AMF (Balivada et al., 2010). A third mouse study assessing the effectiveness of MIONs conjugated to ChL6, an antibody that targets tumor-associated antigen L6, demonstrated significant tumor accumulation and breast cancer tumor growth delays (DeNardo et al., 2007).

**Table 4:** *In vivo* studies of biologically targeted magnetic hyperthermia.

**Reference**	**Field strength (kA/m)**	**Frequency (kHz)**	**Field strength x frequency (A/m•s)**	**Quantity of Fe delivered**	**Target**	**Targeting Mechanism**	**Summary of Results**
Huang (Huang and Hainfeld, 2013)	38 kA/m	980 kHz	3.724 × 10^10^	1700 mg/kg	Squamous Cell Carcinoma	EPR	Durable ablation of tumors in 84% of hyperthermia group compared to 0% for controls
Balivada (Balivada et al., 2010)	5 kA/m	366 kHz	1.830 × 10^9^	13.30 mg/kg^*^	Melanoma	EPR + Porphyrins	Tumor volume was smaller in the hyperthermia group (p < 0.1)
DeNardo (DeNardo et al., 2007)	56–113 kA/m	153 kHz	1.729 × 10^10^	150 mg/kg^*^	Breast Cancer	EPR + Antibody targeting integral membrane glycoprotein	Tumor doubling/tripling/quadrupling times were increased significantly (*p* < 0.05) except for the group that received the lowest energy

* *Assuming 20 g average weight of mice*.

*EPR, enhanced permeability and retention; kA/m, kiloampere/metre; kHz, kilohertz; A/m•s, ampere/meter•second*.

Despite the promising findings outlined above, preclinical studies often apply field strengths, frequencies or quantities of MIONs that are beyond what is clinically feasible and thus, further research is warranted in the areas of design, delivery, and the heating of nanoparticles, to achieve clinical translation in the future. In the following sections, areas requiring further research will be highlighted.

## Factors influencing the efficacy of biologically targeted magnetic hyperthermia

### Field strength and frequency

Achieving and maintaining hyperthermia in the tumor is no easy task. Due to natural thermoregulatory processes, significant power must be delivered to elevate the temperature of a particular region of the body. The heating of MIONs is dependent on a variety of factors including the concentration of MIONs, frequency and the field strength. Currently, the only clinically available AMF system in the world, NanoActivator® (MagForce AG, Germany), can operate at a frequency of 100 kHz and is able to apply fields up to 18 kA/m (Jordan et al., 2001). Although higher frequencies are technically feasible, 100 kHz was chosen to minimize eddy currents and maximize the temperature differential between normal tissues and tumors containing magnetic nanoparticles (Jordan et al., 1993). Eddy currents are electrical currents that are induced within the conductor, in this case the human body, due to the changing magnetic field, as described by Faraday's law of induction. Excessive non-specific heating of normal tissues by eddy currents is the primary determinant of the maximum tolerable field strength and frequency.

At present, there is limited clinical data on the maximum tolerable field strength and frequency. In 1984, Atkinson et al. designed a single-turn induction coil for interstitial magnetic seed therapy and conducted experiments on thoraces of numerous volunteers. They found that field intensities up to 35.8 A/m at a frequency of 13.56 MHz can be tolerated for extended periods of time. Based on this study, the assumption was made that the product of field strength and frequency should not exceed 4.85 × 10^8^ A/m·s (Atkinson et al., 1984). However, this is not an absolute limit and in certain scenarios, this limit may be exceeded (Dutz and Hergt, 2013; Obaidat et al., 2015). In phase 1 and 2 trials of the MagForce system, using lower frequencies of 100 kHz, patients with glioblastoma were able to tolerate up to 13.5 kA/m (1.35 × 10^9^ A/m·s or a median value of 8.5 × 10^8^ A/m·s) whilst patients with prostate cancer were only able to tolerate up to 5 kA/m (5 × 10^8^ A/m·s) due to discomfort in the groin and/or perineal regions (Johannsen et al., 2007a; Maier-Hauff et al., 2007; Nieskoski and Trembly, 2014). This may have been due to boundary effects between tissues of different dielectric constants and conductivity, as well as narrowing of current path in the skin folds such as the groin, resulting in hot spots (Johannsen et al., 2007a). The higher tolerable field strength in patients with glioblastoma is likely to be due to the smaller radius of the head compared to the pelvis or thorax in other studies. Considering that higher field strengths and frequencies will translate to improved heating of tumors, further research is required into improving the tolerable limits of magnetic field strengths and frequencies via improved surface cooling of hotspots that develop in the body, such as the groin (Johannsen et al., 2007b). Furthermore, with shorter duration of treatment, it is possible that higher magnetic field strength or frequency may be achievable. Another possible limitation to the maximum field strength that can be applied clinically relates to the technical challenges of designing and manufacturing a much larger system than the smaller systems utilized in the preclinical studies (Table 4; Jordan et al., 2001). It is advisable that future preclinical studies on biologically targeted magnetic hyperthermia focus on the application of clinically relevant magnetic field strength and frequency of 18 kA/m and 100 kHz currently available on the MagForce system.

Assuming that MIONs have been delivered to the target, the temperature can be adjusted by the alteration of magnetic field strength or frequency. For example, the hyperthermia system from Magforce controls the temperature by adjusting the magnetic field strength. As the effect of hyperthermia is heavily influenced by the temperature reached and for how long this is maintained, it is extremely important to accurately monitor the temperature during therapy and this has been previously achieved with an invasive catheter or specialized software based on imaging (Mahmoudi et al., 2018). Future studies must ensure that hyperthermia is delivered sufficiently by close monitoring of the tumor temperature.

## Dosing and toxicity of magnetic iron oxide nanoparticles

The rate of AMF-induced heating is highly dependent on the concentration of MIONs within the tumor. In clinical trials, up to 31.36 mg of Fe/cm^3^ of tumor, in the form of MIONs, have been administered intra-tumorally in patients with glioblastoma (Maier-Hauff et al., 2011). Feraheme® (AMAG Pharmaceuticals, USA), an FDA approved iron oxide nanoparticle indicated for iron replacement, has been safely delivered intravenously in larger quantity than probably any other FDA approved inorganic nanoparticle so far and the recommended dose is 510 mg of Fe in the form of Feraheme®, followed by a second injection 3 to 8 days later. In the past, several patients have received two additional injections to a total dose of 2.02 g of Fe in the form of Feraheme® within a short period (Lu et al., 2010). In a hypothetical scenario, if 2.02 g of Fe in the form of MIONs, are intravenously administered to a patient with a 35 ml prostate tumor, and assuming that 1% of the dose would reach the tumor, this would result in only about 0.6 mg of Fe/cm^3^ of tumor, far lower than what has been achieved with intra-tumoral administration. In addition, Feraheme contains approximately 3 nm iron oxide cores that are smaller than the MIONs that are typically associated with effective heating (Bullivant et al., 2013). For example, the nanoparticles used by MagForce contain a 12 nm iron oxide core surrounded by aminosilanes and larger crystal cores are likely to be associated with different toxicity profiles. In mice, Huang et al. was able to deliver much higher concentrations of MIONs (5.1 g Fe/kg) and determined an MTD_50_ value of 4.7 g Fe/kg, more than 100 times that delivered per kg in the Feraheme study (Huang and Hainfeld, 2013).

As the interaction of MIONs with their biological environment, and therefore their toxicity, varies with morphology, size, and surface modifications such as the addition of biocompatible coatings and targeting moieties, as well as the route of administration, each formulation needs to be tested thoroughly *in vitro* and *in vivo*. MIONs can mediate toxicity through several mechanisms that all have to be taken into account when evaluating their safety. Most intracellular toxicity is caused by generation of reactive oxygen species whereas *in vivo* disturbances of blood clotting, iron homeostasis and macrophage function, as well as organ toxicities, are additional considerations (Ilinskaya and Dobrovolskaia, 2013; Wu et al., 2014; Wei et al., 2016; Shah and Dobrovolskaia, 2018). A more detailed discussion of MION toxicity can be found in specialized review articles (Reddy et al., 2012; Liu et al., 2013; Arami et al., 2015).

To achieve sufficient heating via intravenous delivery of MIONs, further research is necessary to assess the tolerability of larger quantities of MIONs with bigger cores which are more suited to magnetic hyperthermia, and this will have to be finely balanced with size requirements for efficient intra-tumoral accumulation of nanoparticles.

## Heating efficiency of magnetic iron oxide nanoparticles

In order to minimize the quantity of iron oxide nanoparticles necessary for adequate magnetic hyperthermia, the development of nanoparticles with higher heating efficiency is desirable. The most common parameter for quantifying the heat generated via magnetic induction of MIONs is the Specific Absorption Rate (SAR). The experimental measurement of SAR is relatively simple. It typically involves suspending a known amount of MIONs in a liquid of known heat capacity. The test sample is exposed to an AMF of a specific strength and frequency, and the change in temperature is measured continuously over a period of time. The temperature measurement is carried out with fiber optic temperature probes to avoid electromagnetic interference with the measurement. The SAR is then calculated from the following equation (Kallumadil et al., 2009; Huang et al., 2012):

$$\begin{array}{l} SAR=\frac{C}{m_{np}}\;\left(\frac{dT}{dt}\right){|}_{{\;}_{t=0}} \end{array}$$

where *C* is heat capacity of the fluid per unit mass of fluid, *m*_*np*_ is the mass of magnetic phase suspended in the fluid and *dT/dt* refers to the initial slope of temperature rise *T*, as a function of time, *t*.

It is important to note that SAR is a system-dependent parameter, that is, its value depends on the strength (H) and frequency (f) of the applied magnetic field. Therefore, direct comparison between measurements that are made using different field strength and frequency is not possible. A better parameter for this purpose is the Intrinsic Loss Power (ILP) which is mathematically described by the equation below (Kallumadil et al., 2009):

$$\begin{array}{l} ILP=\frac{SAR}{H^{2}f}=\frac{C}{H^{2}f\;m_{np}}\;\left(\frac{dT}{dt}\right){|}_{{\;}_{t=0}} \end{array}$$

The ILP parameter is introduced under several key assumptions: (1) Test samples are single domain nanoparticles that heat up mainly via rotational relaxation; (2) Magnetic induction systems are of low frequencies at approximately 10^5^–10^6^ Hz; (3) Applied field strength is under the saturation field of the MIONs; (4) For the case of polydisperse MIONs in solution, the crystallite polydispersity index (PDI) has to be greater than 0.1 (Rosensweig, 2002; Kallumadil et al., 2009). If these assumptions are not satisfied, the derived ILP values may not be valid. It is important to note that the published ILPs are only a guide, and the absolute values may not always be reliable due to the variability in the methods used to measure them and given the heating rates are very sensitive to factors such as polydispersity (Gonzales-Weimuller et al., 2009; Wildeboer et al., 2014). Different types of MIONs have highly variable heating properties. Kallumadil et al. found significant variations in the ILP between various commercially available MIONs, ranging from 0.15 to 3.12 nHm^2^/kg. Heating rates can be influenced by several factors such as the ferrous iron content, size, hydrodynamic diameter, shape, number of cores, method of synthesis, and introduction of other metals such as Mn and Zn (Kallumadil et al., 2009; Blanco-Andujar et al., 2015; Hauser et al., 2015; Phong et al., 2017).

Due to the large number of variables, it is difficult to determine precisely how individual factors can impact the heating performance. In addition, the viscosity of the solvent and concentration of MIONs can further dictate the heating properties (Salas et al., 2014). Despite this, there are studies that do provide general insights to the relationship between the various characteristics and the heating properties. Several investigators have shown that in general, larger MIONs are more efficient at generating heat than smaller MIONs. (Gonzales-Weimuller et al., 2009; Lartigue et al., 2011; de La Presa et al., 2012; Jeun et al., 2012). For example, Lartigue et al. produced MIONs ranging from 4 to 35nm and coated them with rhamnose, a type of sugar. When heated under 168 kHz and 21 kA/m, the SAR was 0 W/g of Fe for 4 nm MIONs, 32 W/g of Fe for 10 nm MIONs, 61 W/g of Fe for 16 nm MIONs, and 76 W/g of Fe for 35 nm MIONs (Lartigue et al., 2011).

The shape of the nanoparticle can have a significant influence on the heating performance. Song et al. produced and compared the heating performance of quasi-cubical and spherical Fe_3_O_4_ nanoparticles under 100 kHz and 30 kA/m. Under equal concentration of Fe, the SAR for quasi-cubical nanoparticles were far superior (Song et al., 2012). Another study by Nemati et al. compared deformed cube (octopods) shaped MIONs with spherical nanoparticles of similar volume and demonstrated superior heating performance of the octopods (Nemati et al., 2016). Liu et al. produced ring shaped MIONs (nanorings) and compared the heating performance with a commercial MION called Resovist across a range of magnetic field strengths. Although the difference cannot be entirely attributed to the shape alone due to the differences in size, nanorings demonstrated superior heating performance, especially under the higher ranges of magnetic field strength (Liu et al., 2015). Consequently, magnetic hyperthermia via nanorings resulted in superior tumor control *in vivo* (Liu et al., 2015). Despite the superior heating rates of some of the oddly shaped MIONS, it is important to be aware that the shape can also influence the rate of uptake and toxicity (Hinde et al., 2017). These factors must be considered when designing nanoparticles for clinical applications.

The surface coating can have a significant impact on the heating performance of MIONs. Complete coating of MIONs with a low heat conductor such as SiO_2_ shell can prevent the outflow of heat and reduce the heating efficiency (Gonzalez-Fernandez et al., 2009; Rivas et al., 2012). Furthermore, the thickness of the coating can also impact the heating efficiency. Liu *et al*. coated MIONs with polyethylene glycol (PEG) polymer of various length ranging from 2,000 to 20,000 Da and found that MIONs coated with shorter polymers generally heat better, possibly due to increased Brownian loss, improved thermal conductivity and dispersibility (Liu et al., 2012). One exception to this was the 31 nm MION which heated better when coated with longer PEG polymers. This was ascribed to potential agglomeration of the 31 nm MIONs with the shorter PEG, highlighting a delicate balance between stability and heating performance. The coating can also influence the pharmacokinetics of MIONs in the body which is an important consideration when developing MIONs for hyperthermia (Arami et al., 2015). Doping MIONs with Mg or Zn is another strategy that has resulted in nanoparticles with superior heating profiles, resulting in better tumor control *in vivo* (Jang et al., 2009).

Interestingly, one of the highest ILPs (23.41 nHm^2^/kg) to have been reported in the past was on bacterially derived MIONs, which have a mean core diameter of approximately 30 nm (Hergt et al., 2005). Bacterial magnetosome-like cubic nanoparticles were later produced by Martinez-Boubeta et al. and demonstrated superior heating efficiency compared to spheroidal MIONs of similar size (Martinez-Boubeta et al., 2013). Le Fevre et al. have evaluated the effectiveness of magnetic hyperthermia via intra-tumorally delivered magnetosomes and achieved superior tumor control compared to chemically synthesized MIONs (Le Fèvre et al., 2017). Recently, Sangnier et al. demonstrated that magnetosomes can be tagged with tumor targeting peptide, arginine-glycine-aspartic acid (RGD), then administered intravenously in mice models for targeted delivery to tumors (Plan Sangnier et al., 2018). They applied photothermal therapy rather than magnetic hyperthermia as it was thought to be more effective. However, such approaches are likely to be limited for deep seated tumors in humans and thus, further work is required to evaluate its application for magnetic hyperthermia. Many other types of nanoparticles have been produced in the past for magnetic hyperthermia and more details can be found in other specialized review articles (Blanco-Andujar et al., 2017; Hedayatnasab et al., 2017). Higher heating efficiency would be highly desirable as it would reduce the quantity of nanoparticles, field strength and frequency required to induce significant heating.

## Targeting of MIONs

Intravenously administered nanoparticles preferentially accumulate within tumors owing to their leaky vasculature and poor drainage. This EPR effect is well documented and was recently demonstrated in human tumors (Clark et al., 2016). In addition, structural and surface modification of MIONs can further increase tumor accumulation and up to ~15.5%ID/g have been reported in the past (Xu et al., 2016).

Targeting of cancer cells with antibodies or other ligands can further improve the accumulation of nanoparticles within the tumor. MIONs conjugated to antibodies have been previously delivered to several tumor specific antigens including L6, HER-2 and PSMA for medical imaging and magnetic hyperthermia (DeNardo et al., 2007; Zhang et al., 2011; Tse et al., 2015). As mentioned earlier, one of the best examples is a study by DeNardo et al. in which MIONs conjugated to ChL6, an antibody that targets tumor-associated antigen L6, demonstrated significant tumor accumulation and breast cancer tumor growth delays under an AMF (DeNardo et al., 2007). Despite the potential for enhanced delivery, targeting can be associated with significant challenges in terms of the chemistry of conjugation and stability of ligand or antibody bound to nanoparticles. For example, MLN2704, a prostate specific antigen directed immunoconjugate for delivering chemotherapeutics to prostate cancer was associated with significant toxicity and limited activity due to deconjugation of the targeting antibody once in circulation (Milowsky et al., 2016). In a clinical trial of CALAA-01, a ligand bound nanoparticle siRNA delivery system, 21% of patients discontinued the study due to an adverse event and it was proposed that ligand instability was responsible for the undesirable toxicity (Zuckerman and Davis, 2015). Some of these limitations can be overcome by the application of bispecific antibodies that can spontaneously bind to both the poly ethylene glycol (PEG) coated nanoparticles and cancer specific antigens such as prostate specific membrane antigen (PSMA) or epidermal growth factor receptor (EGFR). Bispecific antibodies are composed of 2 separate single-chain fragment (scFv) and are smaller than whole antibodies. It can be stored in the freezer separate to the nanoparticles, thereby overcoming the stability issue. When administered with any PEGylated nanoparticles prior to or at the time of delivery, bispecific antibodies will spontaneously associate itself with PEGylated nanoparticles. Within the tumor, bispecific antibodies will bind to cancer specific antigens and keep the nanoparticle in close proximity to the target cancer cells, thereby, enhancing tumor accumulation (Howard et al., 2016).

To overcome the limitations of antibodies, MIONs can alternatively be conjugated to cancer specific peptides, glycosaminoglycans or aptamers. In order to target ovarian cancer, Taratula et al. synthesized MIONs conjugated to an ovarian cancer targeting Luteinizing Hormone-Release Hormone (LHRH) peptide. *In vitro*, LHRH peptide coating improved the ability of MIONs to associate with ovarian cancer cells and resulted in a significant reduction in cell viability under an alternating magnetic field (Taratula et al., 2013). For reduced immunogenicity, MIONs can be coated with hyaluronic acid, a biocompatible material that is naturally found in our body. Hyaluronic acid can target cancer cells via CD44 receptor, a commonly found cell surface marker in epithelial tumors and its potential role in magnetic hyperthermia has been demonstrated *in vitro* (Thomas et al., 2015). Nair et al. produced glioma targeting aptamers, composed of oligonucleotides, for conjugation with dextran coated iron oxide nanoparticles. Using the targeted MIONs, they were able to induce preferential damage to glioma cells via mechanical oscillation induced by a rotating magnetic field (Nair et al., 2010). It is possible that such nanoparticles may be applied for magnetic hyperthermia in the future.

For further enhancement of hyperthermia, MIONs can be directed toward intracellular organelles of cancer cells via conjugation of organelle targeting peptides. Peng et al. administered transferrin and nuclear targeting TAT peptide conjugated MIONs to mice and applied photothermal hyperthermia (Peng et al., 2017). When compared to transferrin conjugated MIONs, nuclear targeting MIONs demonstrated significant improvement in tumor control (Peng et al., 2017). Additional studies are required to confirm that such intracellular targeting strategies may be applicable for magnetic hyperthermia. Despite these exciting approaches to targeting, there is multitude of factors that can influence its effectiveness and a detailed evaluation can be found in specialized reviews (Rosenblum et al., 2018).

Another novel approach to improved tumor targeting is to suppress the reticuloendothelial system with drugs prior to the delivery of MIONs. For example, Abdollah et al. demonstrated that the suppression of Kupffer cells in the liver with dextran sulfate can significantly increase the circulating half-life of non-targeted MIONs by inhibiting the liver uptake (Abdollah et al., 2014). It is uncertain whether dextran sulfate suppression can also be applied in combination with ligand- or antibody-conjugated MIONs to prevent liver uptake and further research is warranted in this area.

Overall, several strategies are being evaluated in order to effectively target nanoparticles to the tumor whilst sparing normal tissue. Improved targeting will ultimately be the key to delivering sufficient quantities of MIONs for selective heating of tumors.

## Magnetically targeted MIONs

Due to their magnetic properties, MIONs can be directed toward the tumor via a magnetic field. This can be applied in combination with targeted MIONs for effective magnetic hyperthermia. There are several notable examples of this approach. For gene therapy, MIONs have been used to direct intravenously administered silencing RNAs toward gastric tumors in mouse models under a magnetic field (Namiki et al., 2009). In a separate study, Garcia-Jimeno et al. were able to direct magnetoliposomes, with the aid of a magnetic field, toward the target and away from the liver and the spleen of mice (García-Jimeno et al., 2012).

For maximum uptake and retention in the tumor, it is important for MIONs or other nanoparticles to extravasate and reach the cancer cells. This can be achieved by disrupting the endothelial barrier with an external magnetic field. Qui et al. injected MIONs into mouse tail vein and used an external magnetic field to direct the particles into the lateral tail vein. Histological examination revealed that MIONs accumulated in the endothelial tissue. When a fluorophore was injected systemically, fluorescence signal was higher in the tail of the mice subjected to the magnetic field and MIONs, due to a disruption of endothelial lining (Qiu et al., 2017). Combining these approaches may potentially improve the therapeutic efficacy of magnetic hyperthermia in the future.

## Other methods to improve the impact of targeted magnetic heating

Other novel methods of improving the effectiveness of magnetic hyperthermia have been explored. Espinosa et al. applied near-infrared laser irradiation (808 nm) during magnetic hyperthermia *in vivo* and demonstrated 2–5 fold improvements in heating when compared to magnetic hyperthermia alone although such approaches would be limited to surface tumors owing to the poor tissue penetration of laser irradiation (Espinosa et al., 2016).

In the past, there have been attempts to biologically enhance the effectiveness of magnetic hyperthermia with hyperthermia enhancing drugs such as the heat shock protein (HSP) 90 inhibitor Geldanomycin. When cells are heated, HSP 90 plays a key role in stabilizing proteins, thus, limiting the downstream effects of protein denaturation. Therefore, the inhibition of HSP 90 can lead to improved effectiveness of hyperthermia and reduce thermotolerance. For example, Ito et al. delivered Geldanomycin, and applied magnetic hyperthermia in a mouse melanoma model, which resulted in significant improvement in tumor control when compared to magnetic hyperthermia alone (Ito et al., 2009). This approach is particularly promising as HSP 90 inhibitors can independently enhance the effectiveness of radiotherapy, even in the absence of hyperthermia (Schilling et al., 2015).

There are many other hyperthermia enhancers that have been reported in the past but the majority of these agents have not been evaluated in combination with magnetic hyperthermia (Marchal et al., 1986). Protease inhibitors are another class of potent hyperthermia enhancers that have been evaluated *in vitro*. It is thought that the enhancement is achieved by inhibiting the clearance of denatured proteins within the cells (Zhu et al., 1995).

Another novel strategy is to combine magnetic hyperthermia with thermally sensitive liposomes. This can be achieved by creating a liposome with magnetic iron oxide cores embedded within. When an AMF is applied, the magnetic nanoparticles will trigger the release of the lipososomal contents. As AMF can be applied to a specific region of the body, this could result in targeted drug release and improved therapeutic effectiveness. For example, Yang et al. produced a CD90 targeted magnetoliposome encapsulating 17-AAG, a HSP 90 inhibitor. The magnetoliposome was able to simultaneously heat liver cancer stem-like cells and trigger the release of 17-AAG, thereby improving the effectiveness of magnetic hyperthermia (Yang et al., 2015).

## Conclusion

Hyperthermia can lead to cell death via modulation of various cellular processes and is an effective treatment that can enhance the outcomes of radiotherapy and chemotherapy. One of the disadvantages is the lack of specificity toward malignant cells compared to healthy tissue. Systemic administration of targeted MIONs has the potential to improve the specificity of hyperthermia and improve its efficacy. However, several limitations must be resolved before this technology can progress to clinic. Future preclinical studies should focus on designing MIONs that can target and heat tumors more effectively. Furthermore, various hyperthermia enhancers should be evaluated in combination with magnetic hyperthermia, with the ultimate objective of achieving clinical feasibility.

## Author contributions

DC drafted the manuscript, drew the figures and constructed the tables. DC, ML, JG, RQ, YN, FM, MJ, TD, and MK discussed the outline and critically reviewed the paper, the content, and the figures used.

### Conflict of interest statement

The authors declare that the research was conducted in the absence of any commercial or financial relationships that could be construed as a potential conflict of interest.
